# A review and meta-analysis of prospective studies of red and processed meat, meat cooking methods, heme iron, heterocyclic amines and prostate cancer

**DOI:** 10.1186/s12937-015-0111-3

**Published:** 2015-12-21

**Authors:** Lauren C. Bylsma, Dominik D. Alexander

**Affiliations:** EpidStat Institute, 2100 Commonwealth Blvd, Suite 203, Ann Arbor, MI USA; EpidStat Institute, Bothell, WA USA

**Keywords:** Prostate cancer, Red meat, Processed meat, Meta-analysis, Review, Epidemiology, Cooking methods, Heterocyclic amines, Diet

## Abstract

Prostate cancer remains a significant public health concern among men in the U.S. and worldwide. Epidemiologic studies have generally produced inconclusive results for dietary risk factors for prostate cancer, including consumption of red and processed meats. We aimed to update a previous meta-analysis of prospective cohorts of red and processed meats and prostate cancer with the inclusion of new and updated cohort studies, as well as evaluate meat cooking methods, heme iron, and heterocyclic amine (HCA) intake exposure data. A comprehensive literature search was performed and 26 publications from 19 different cohort studies were included. Random effects models were used to calculate summary relative risk estimates (SRREs) for high vs. low exposure categories. Additionally, meta-regression analyses and stratified intake analyses were conducted to evaluate dose-response relationships. The SRREs for total prostate cancer and total red meat consumption, fresh red meat consumption, and processed meat consumption were 1.02 (95 % CI: 0.92–1.12), 1.06 (95 % CI: 0.97–1.16), and 1.05 (95 % CI: 1.01–1.10), respectively. Analyses were also conducted for the outcomes of non-advanced, advanced, and fatal prostate cancer when sufficient data were available, but these analyses did not produce significant results. No significant SRREs were observed for any of the meat cooking methods, HCA, or heme iron analyses. Dose-response analyses did not reveal significant patterns of associations between red or processed meat and prostate cancer. In conclusion, the results from our analyses do not support an association between red meat or processed consumption and prostate cancer, although we observed a weak positive summary estimate for processed meats.

## Introduction

Prostate cancer is the second most common cancer in men worldwide, with an estimated 1.1 million incident cases and 0.3 million deaths occurring in 2012, according to the World Health Organization (WHO) Cancer Report 2014 [1]. Prostate cancer is more commonly diagnosed in high-resource countries, which is likely attributable to higher age attainment and the availability and prevalence of prostate-specific antigen (PSA) testing. Incidence rates in these countries have generally leveled off in the past two decades, but continue to increase in low and middle resource countries [2]. In the United States (U.S.), an estimated 220,800 new cases and 27,540 deaths due to prostate cancer will occur in 2015 [3]. Prostate cancer comprises 13.3 % of all new cancer cases and 4.7 % of cancer deaths in the U.S. The National Cancer Institute has published that approximately 14.0 % of men in the U.S. will be diagnosed with prostate cancer at some point during their lifetime, although the number of incident cases has decreased by an average of 4.3 % each year from 2002 to 2012 [3].

Major identified risk factors for prostate cancer of clinical significance include increasing age, family history of this malignancy, and African-American race [1]. Epidemiologic studies have demonstrated that the disparity in incidence and mortality rates observed in low resource countries tends to disappear after migration to a high resource country, suggesting that lifestyle factors may influence the risk of prostate cancer [4, 5]. Many dietary factors have been evaluated for prostate cancer risk, including consumption of dairy products, alcohol, vitamin E, animal fat, and lycopene, with generally inconclusive results [6]. Specifically, several epidemiologic studies have assessed the relationship between consumption of red and processed meats and risk of prostate cancer. In their 2014 review of prostate cancer risk factors, the World Cancer Research Fund/American Institute for Cancer Research (WCRF/AICR) found the available epidemiologic and mechanistic data for red and processed meats and prostate cancer to be limited and insufficient to make a conclusion in relation to prostate cancer risk [7].

A postulated hypothesis for a potential association between meat consumption and prostate cancer is the presence of heterocyclic amines (HCA) that are formed in cooked meats, particularly meats cooked at high temperatures or to a “well-done” degree [8–10]. Other compounds suspected to be associated with increased cancer risk are polycyclic aromatic hydrocarbons (PAH), particularly benzo(a)pyrene (B(a)P), also formed by meats cooked over flame or at high temperature, and heme iron, a compound found predominantly in red meat but also found in poultry and fish [10, 11]. However, assessing exposures such as HCA or heme iron in epidemiologic studies is not a straightforward process as these are factors not ascertained via food frequency questionnaires, and thus, are evaluated as secondary exposures.

We published a meta-analysis in 2010 of 15 prospective studies of red and processed meat intake and prostate cancer and observed no association for red meat consumption and a weakly elevated summary association between processed meat intake and prostate cancer [12]. As updated cohorts and new studies have since been published [13–19], the state of the epidemiologic science was updated in the current review and meta-analysis. Thus, our specific objectives were as follows: (i) calculate summary relative risk estimates for red and processed meat intake and prostate cancer, (ii) conduct subgroup analyses for red and processed meats and advanced, non-advanced, and lethal prostate malignancy, (iii) conduct dose-response and meta-regression analyses, (iv) identify sources of heterogeneity through sub-group and sensitivity analyses, and (v) assess the potential for publication bias in the literature for red and processed meat and prostate cancer. Furthermore, to our knowledge, no meta-analysis has been published on prospective studies that assessed exposure to HCAs, PAHs from cooking, heme iron, or data on meat cooking methods and risk of prostate cancer. Therefore, additional objectives for this meta-analysis were to estimate summary relative risk estimates for these compounds and risk of prostate cancer.

## Review

**Fig. 1:**
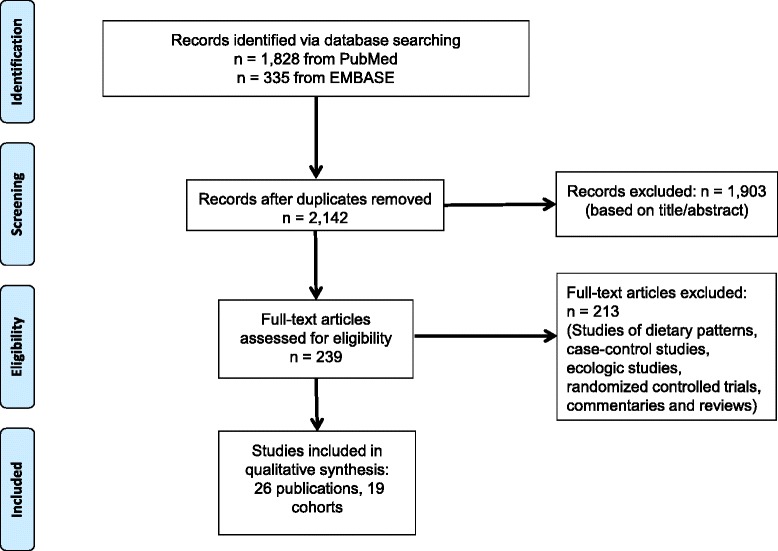
PRISMA diagram. Systematic search for eligible studies of red and processed meat consumption and prostate cancer

We followed the Preferred Reporting Items for Systematic Reviews and Meta-Analyses (PRISMA) guidelines for this systematic review and meta-analysis [20]. The PRISMA checklist was included as part of the submission process. The 27 checklist items pertain to the content of a systematic review and meta-analysis, which includes the title, abstract, methods, results, discussion, and funding. A diagram of the search strategy is shown in Fig. 1.

### Literature search and study identification

Literature searches were conducted to identify relevant articles in the PubMed and EMBASE databases through September, 2015 with no lower date limit. The exposures of interest included fresh red meat (generally defined as beef, pork, or lamb, e.g. steak, pork chops, etc.), processed meat (typically defined as beef, pork, or lamb that has undergone methods of preservation, including smoking, curing, or drying, e.g. bacon, hot dogs, etc.; some studies may have included processed poultry, e.g., deli meats, turkey hot dogs, etc.), and total red meat (a combination of fresh red meat and processed meat) [21, 22]. Additional exposures of interest included various methods of cooking or consuming meat (grilled, fried, broiled, well-done, rare), heterocyclic amines (DiMeIQx, MeIQx, PhIP, total mutagenic activity), B(a)P, and heme iron. We did not include data on poultry or fish, apart from their possible inclusion in the processed meat variables. The outcomes of interest included total prostate cancer, non-advanced prostate cancer (Stage I/II), advanced prostate cancer (Stage III/IV), and fatal prostate cancer. The following terms were used in the search string: “prostate cancer”[Title/Abstract] AND (“meat”[Title/Abstract] OR “foods”[Title/Abstract] OR “food habits”[Title/Abstract] OR “feeding behavior”[Title/Abstract] OR “food preferences”[Title/Abstract] OR “red meat”[Title/Abstract] OR “minced meat”[Title/Abstract] OR “ham”[Title/Abstract] OR “bacon”[Title/Abstract] OR “sausage”[Title/Abstract] OR “processed meat”[Title/Abstract]) OR (“heterocyclic amines”[Title/Abstract] OR “polycyclic aromatic hydrocarbons”[Title/Abstract] OR “heme iron”[Title/Abstract] OR “cooked meat”[Title/Abstract] OR “cooking methods”[Title/Abstract] OR “grilled meat”[Title/Abstract] OR “charred meat”[Title/Abstract]). Additionally, the bibliographies of relevant meta-analyses and reviews were hand searched to capture studies not identified through our electronic searches.

We excluded case–control studies from this analysis because of the abundance of available prospective cohort studies and the documented issues that accompany nutritional assessments in case–control studies of diet and cancer [23]. We also excluded studies of dietary patterns unless the authors analyzed an exposure for red or processed meat independently of a dietary pattern. In addition, cross-sectional surveys, ecologic analyses, case reports, editorials, and experimental animal studies were excluded. If multiple articles were identified that analyzed data within the same cohort, analyses presented from different studies that utilized unique exposures or outcomes (e.g. one study reporting fatal prostate cancer cases only and another study reporting total prostate cancer) were included in the meta-analysis. For example, in a sensitivity analysis by race, we used data from Major et al. 2011 (NIH-AARP cohort) for African-American men, although data from the full cohort (Sinha et al. 2009) was used for overall analyses. If no differences in analyses were noted between published studies of the same cohort, the decision of which data to include was made using these criteria: (i) size of the cohort, (ii) number of years of follow-up, and (iii) number of confounders adjusted for in multivariate models.

### Data extraction and statistical analysis

The following data were extracted from each study: author, year of publication, cohort name, description of cohort, size of analytic cohort, years of follow-up, race, outcome, number of incident cases, year diet was assessed, diet assessment method, meat/cooking method/HCA definition and items included in definition, exposure category, exposure category definition and units, RR, 95 % Confidence Interval (CI), *p*-value for trend, and statistical adjustments. If both crude and adjusted RRs were presented within a study, the most fully adjusted RR was abstracted.

Random effects models were used to calculate summary relative risk estimates (SRRE), 95 % CIs, *p*-values for heterogeneity, and I^2^ values. This model creates study weights that are equal to the inverse of the variance of each study’s effect estimate according to the methodology propounded by DerSimonian and Laird [24]. Relative risks comparing the highest unit of consumption against the lowest unit of consumption within each study were combined across all studies to produce a summary association. Fixed effects models were used to combine mutually-exclusive results within individual studies if no composite variable for total red meat, fresh red meat, or processed meat was provided (e.g. combining results for bacon, sausage, and salami into one measure of “processed meat”), so that one estimate for each meat category was included in each model. Primary meta-analysis models were created for the exposures of total red meat, fresh red meat, and processed red meat, and for the outcomes of total prostate cancer, non-advanced prostate cancer, advanced prostate cancer, and fatal prostate cancer, if sufficient data (≥2 studies) were available. Secondary meta-analysis models were generated to evaluate the exposures of various cooking methods, HCA, B(a)P, and heme iron. Sub-group and sensitivity analyses were performed to evaluate potential patterns of associations and sources of heterogeneity by descriptive study factors such as race, geographic region, and number of cases. Sensitivity analyses removing one study at a time were conducted to determine the effect of each study on the model.

As the units of consumption varied across studies, we harmonized the data into “servings per day” based on U.S. Department of Agriculture reference amounts customarily consumed per eating occasion: 85 grams per one serving of total red meat, 75 grams per serving of fresh red meat, and 50 grams per serving of processed red meat [25]. Studies that provided intake categories of grams per 1000 kcal were doubled to estimate intake for a typical 2000 kcal diet. We assessed dose-response relationships between red and processed meat and prostate cancer using two different methods: linear regression modelling and categorical analyses. We first conducted meta-regression analyses to estimate the risk of prostate cancer per each incremental serving of red or processed meat intake. To do this, we included all risk estimates and 95 % confidence intervals for each intake category reported in the individual studies. As a complimentary dose-response analysis, we performed meta-analyses based on stratified intake categories utilizing relative risks from every reported intake category. Thus, we were able to incorporate all reported intake stratifications at the individual study level, and the meta-analytic intake groups were created after visualizing the distribution of the exposure data across all studies. However, this method is restricted to only those studies that report quantitative intake metrics, thereby excluding studies that report RRs for unspecified levels of intake, such as low, medium, or high, or undefined quantiles of intake.

The percentage of variance due to between-study heterogeneity was estimated using the Cochran’s Q test and I^2^ statistic [26]. Publication bias was evaluated visually by creating funnel plots, as well as by conducting Egger’s regression tests and using the Duval and Tweedie imputation method [27]. Forest plots were generated for total red meat consumption and total prostate cancer, processed meat and total prostate cancer, and HCA intake and total prostate cancer. All analyses were done using Comprehensive Meta-Analysis Software (version 3.3.070; Biostat, Englewood, New Jersey, USA).

## Results

We identified 26 prospective cohort studies of red and processed meat consumption and prostate cancer from 19 different cohorts. Descriptive study characteristics of prospective cohort studies of red and processed meat consumption and prostate cancer risk are presented in Table 1 [13–15, 19, 28–45]. The majority of the cohorts (fourteen) were conducted in the United States, one in Canada, one in Japan, and three were conducted in Europe. Over 700,000 male participants were evaluated in this meta-analysis. The duration of follow-up ranged from 6 to 22 years. Although several studies were not performed in the United States, the amount of red and processed meat consumed was generally comparable to levels consumed in the U.S. diet, according to the USDA [46]. Table 2 presents the results of the meta-analyses for prostate cancer and intake of total red meat, fresh red meat, and processed meat.

**Table 1:** Characteristics of prospective cohort studies of red and processed meat and prostate cancer ^a^

Author, year	Study cohort (Country)	No. of subjects (No. of cases)	Age range (mean)	Year baseline diet assessed	Follow up, years	Meat exposures analyzed	Prostate cancer outcome	Statistical adjustments
Agalliu et al, 2011 [13]	Canadian Study of Diet, Lifestyle and Health (Canada)	1864 (661)	69.3 (10.5) [mean (SD) controls]; 66.2 (8.4) [mean (SD) cases]	1995–1998	8	Red meat	All PCa; nonadvanced PCa; advanced PCa	Age at baseline, race, BMI, exercise activity, and education
Allen et al, 2004 [29]	Life Span Study Cohort (Japan)	18,119 (196)	51–89	1979–1980	16.9 [mean]	Pork	All PCa	Age, calendar period, city of residence, radiation dose and education level
Allen et al, 2008 [30]	EPIC (Europe)	142,241 (2727)	52 [median]	1989–2004	18	Red meat; processed meat	All PCa	Study center, education, marital status, height, weight, and energy intake
Cross et al, 2005 [47]	PLCO (US)	29,361 (1338)	55–74	1993–2001	11	Red meat; processed meat	Total PCa; incident PCa; advanced PCa	Age, race, study center, fam hx, history of diabetes, number of screening exams during follow-up, smoking status, physical activity, aspirin use, BMI, and intake of total energy, supplemental vitamin E, and lycopene
Gann et al, 1994 [32]	PHS (US)	240 (120)	40–84	1982	6	Beef, pork, or lamb as a main dish	All PCa	None reported
Hsing et al, 1990 [33]	LBC (US)	17,633 (149)	≥35	1966	20	Red meat (processed and unprocessed)	Fatal PCa	Age, tobacco use
Koutros et al, 2008 [34]	AHS (US)	23,080 (668)	48.3 [mean]	1993–1997	10	Red meat; pork chops/ham steaks; beef steaks; hamburgers; bacon/sausage	Total PCa; incident PCa; advanced PCa	Age, state of residence, race, fam hx, and smoking status
Le Marchand et al, 1994 [35]	Hawaiian State Department of Health (US)	8881 (198)	≥18	1975–1980	14	Pork; beef	Total PCa; localized stage PCa; regional and distant stage PCa	Age, ethnicity, and income
Major et al, 2011 [19]	NIH-AARP (US)	7949 (1089) (Blacks only)	50–71	1995–1996	11	Red meat; processed meat	Total PCa; Advanced PCa	Age, education, marital status, fam hx, hx of diabetes, smoking, health status, BMI, alcohol, fruit intakes
Michaud et al, 2001 [36]	HPFS (US)	47,780 (1987)	40–75	1986	10	Red meat; beef, pork, lamb (main dish); beef, pork, lamb (sandwich or mixed dish); hamburger, processed meat (sausage, salami, bologna); bacon; hot dogs	Total PCa, Advanced PCa	Age, calories, calcium, smoking, tomato use, vigorous exercise, saturated and alpha linolenic fat
Mills et al, 1989 [37]	Seventh Day Adventists (US)	14,000 (180)	≥25	1976	6	Beef hamburger; beef steak; other beef and veal; beef index	All PCa	Age
Neuhouser et al, 2007 [38]	CAROT (US)	12,000 (890)	50–69	1989	11 [mean]	Red meat	Total PCa; aggressive PCa; nonaggressive PCa	Age, energy intake, BMI, smoking, fam hx, and race/ethnicity
Park et al, 2007 [39]	Multiethnic Cohort (US)	82,483 (4404)	≥45	1993–1996	8	Red meat; beef; pork; processed meat	Total PCa; non-localized or high grade PCa;	Time on study, ethnicity, family history of prostate cancer, education, BMI, smoking status and energy intake
Richman et al, 2011 [15]	HPFS (US)	27,607 (199)	40–75	1986	22	Total red meat (processed and unprocessed); unprocessed red meat; processed red meat	Lethal PCa	Age, energy, BMI, smoking, vigorous activity, and lycopene intake
Rodriguez et al, 2006 [40]	Cancer Prevention Study II (US)	65,590 (5113)	50–74	1992	9	Total red meat (processed and unprocessed); unprocessed red meat;	All PCa; metastatic PCa	Age at entry, total calorie intake, BMI, level of education, fam hx, history of PSA testing, and hx of diabetes
Rohrmann et al, 2007 [41]	CLUE II (US)	3892 (199)	≥35	1989	15	Red meat (processed and unprocessed); beef; pork; processed meats; sausages; bacon; ham/lunchmeat; hot dogs	Total PCa; low-stage PCa; high-stage PCa	Age, energy intake, consumption of tomato products, BMI at age 21, and intake of saturated fat
Schuurman et al, 1999 [42]	NLCS (Netherlands)	2167 (642)	55–69	1986	6.3	Beef; pork; minced meat (beef and pork); boiled ham; bacon	Total PCa; localized tumors; advanced tumors	Age, fam hx, and socioeconomic status
Severson et al, 1989 [43]	Hawaiian Men of Japanese Ancestry (US)	7998 (174)	46–65	1965–1968	21	Ham, bacon, sausage	Total PCa	Age
Sinha et al, 2009 [44]	NIH-AARP (US)	175,343 (10,313)	50–71	1995–1996	8	Red meat (processed and unprocessed); processed meat	Total PCa; advanced PCa; fatal PCa	Age, total energy intake, race/ethnicity, education, marital status, undergoing prostate-specific antigen testing in the past 3 years, hx of diabetes, BMI, smoking history, frequency of vigorous physical activity, and intakes of alcohol, calcium, tomatoes, alpha-linolenic acid, vitamin E, zinc, and selenium
Veierod et al, 1997 [45]	Norwegian men 1977–1983 (Norway)	25,708 (72)	16–56	1977–1983	15	Main meals with hamburgers, meatballs, etc.	Total PCa	Age at inclusion and attained age
Wright et al, 2012 [14]	ATBC (Finland)	27,111 (1929)	50–69	1985–1988	21	Red meat; beef; sausages	All PCa; advanced PCa	Age, energy intake, smoking dose and duration, trial intervention assignment, education level, and dietary fat intake
Wu et al, 2006 [28]	HPFS	47,725 (3002)	40–75	1986	14	Total red meat, processed meat	Advanced PCa	Age, height, smoking, family history of prostate cancer, race, history of vasectomy, vigorous exercise, body mass index, alcohol intake, and total energy intake

^a^ PCa, prostate cancer; BMI, body mass index; fam hx, family history; ATBC, Alpha-Tocopherol Beta-Carotene Cancer Prevention Study; EPIC, European Prospective Investigation into Cancer and Nutrition; PLCO, Prostate, Lung, Colorectal, Ovarian Screening Trial; PHS, Physicians’ Health Study; LBC, Lutheran Brotherhood Cohort; AHS, Agricultural Health Study; NIH-AARP, National Institutes of Health-American Association for Retired Persons Diet and Health Study; HPFS, Health Professionals Follow-up Study; CAROT, Carotene and Retinol Efficacy Trial; NLCS, Netherlands Cohort Study

**Table 2:** Summary relative risk estimates (SRRE), 95 % confidence intervals (CI), *p*-values for heterogeneity and I^2^ statistics for red and processed meat intake and prostate cancer (high vs. low exposure unless otherwise noted)

Model (number of studies ^a^ or data points^†^)	SRRE	95 % CI	*P*-value for Heterogeneity; I^2^
Total red meat			
Total red meat and total prostate cancer (*n* = 10)	1.02	0.92–1.12	0.006; 61.0 %
*Per 1 serving/day increase*	0.96	0.91–1.00	
*Intake of 0–0.50 servings/day (n = 5)*	1.41	1.09–1.81	0.189; 34.85 %
*Intake of >0.50–1.0 servings/day (n = 13)*	1.02	0.98–1.05	0.735; 0.00 %
*Intake of >1.0–1.5 servings/day (n = 10)*	1.01	0.93–1.10	0.077; 42.07 %
*Intake of >1.0 servings/day (n = 7)*	0.88	0.75–1.04	0.005; 67.68 %
Total red meat and non-advanced prostate cancer (*n* = 3)	0.98	0.63–1.53	0.051; 66.3 %
*Per 1 serving/day increase*	0.71	0.46–1.10	
*Intake of <1 serving/day (n = 4)*	1.10	0.92–1.31	0.619; 0.00 %
*Intake of >1 serving/day (n = 5)*	0.89	0.66–1.19	0.060; 55.69 %
Total red meat and advanced prostate cancer (*n* = 9)	1.01	0.86–1.17	0.177; 30.2 %
*Per 1 serving/day increase*	0.97	0.91–1.04	
*Intake of 0–0.75 servings/day (n = 9)*	1.08	0.94–1.23	0.319; 13.78 %
*Intake of >0.75–1.0 servings/day (n = 4)*	1.06	0.92–1.23	0.697; 0.00 %
*Intake of >1.0–1.5 servings/day (n = 8)*	1.04	0.92–1.18	0.481; 0.00 %
*Intake of >1.0 servings/day (n = 4)*	1.14	0.95–1.37	0.328; 12.95 %
Total red meat and fatal prostate cancer (*n* = 3)	1.06	0.82–1.37	0.35; 4.80 %
*Per 1 serving/day increase*	1.05	0.73–1.52	
*Intake of <1 serving/day (n = 5)*	1.09	0.91–1.30	0.350; 9.89 %
*Intake of >1 serving/day (n = 5)*	1.15	0.96–1.38	0.474; 0.00 %
Fresh red meat			
Fresh red meat and total prostate cancer (*n* = 9)	1.06	0.97–1.16	0.113; 38.3 %
*Per 1 serving/day increase*	0.91	0.81–1.03	
*Intake of 0- < 0.25 servings/day (n = 9)*	1.08	0.97–1.19	0.924; 0.00 %
*Intake of 0.25- < 0.5 servings/day (n = 8)*	1.07	1.02–1.13	0.519; 0.00 %
*Intake of 0.5–0.75 servings/day (n = 6)*	1.04	0.97–1.12	0.363; 8.28 %
*Intake of >0.75 servings/day (n = 9)*	1.07	0.95–1.19	0.171; 30.95 %
Fresh red meat and advanced prostate cancer (*n* = 6)	1.01	0.86–1.20	0.180; 34.107 %
*Per 1 serving/day increase*	0.88	0.69–1.12	
*Intake of 0–0.25 servings/day (n = 9)*	1.14	0.97–1.34	0.776; 0.00 %
*Intake of >0.25–0.50 servings/day (n = 8)*	0.99	0.89–1.10	0.543; 0.00 %
*Intake of >0.50 servings/day (n = 10)*	0.99	0.89–1.11	0.398; 4.66 %
Processed meat			
Processed red meat and total prostate cancer (*n* = 11)	1.05	1.01–1.10	0.406; 3.38 %
*Per 1 serving/day increase*	1.01	0.96–1.05	
*Intake of 0–0.25 servings/day (n = 9)*	1.05	0.99–1.10	0.369; 7.93 %
*Intake of >0.25–0.50 servings/day (n = 12)*	1.05	1.00–1.09	0.407; 3.91 %
*Intake of >0.50–0.75 servings/day (n = 6)*	1.02	0.97–1.07	0.506; 0.00 %
*Intake of >0.75 servings/day (n = 10)*	1.04	0.99–1.10	0.176; 29.20 %
Processed meat and advanced prostate cancer (*n* = 8)	1.12	0.95–1.33	0.040; 52.4 %
*Per 1 serving/day increase*	1.10	0.98–1.24	
*Intake of 0–0.25 servings/day (n = 12)*	0.95	0.80–1.12	0.004; 60.09 %
*Intake of >0.25–0.50 servings/day (n = 9)*	1.07	0.90–1.26	0.009; 60.61 %
*Intake of >0.50 servings/day (n = 8)*	1.13	0.99–1.29	0.116; 39.42 %
Processed meat and fatal prostate cancer (*n* = 2)	1.09	0.63–1.90	0.073; 68.9 %
*Per 1 serving/day increase*	0.84	0.44–1.61	
*Intake of <0.50 servings/day (n = 4)*	0.93	0.75–1.15	0.276; 22.49 %
*Intake of >0.50 servings/day (n = 3)*	0.95	0.69–1.30	0.112; 54.36 %

^a^ Number of studies indicated in parentheses for high vs. low analyses; ^†^number of data points indicated in parentheses for dose- response analyses (in italics)

### Total red meat

**Fig. 2:**
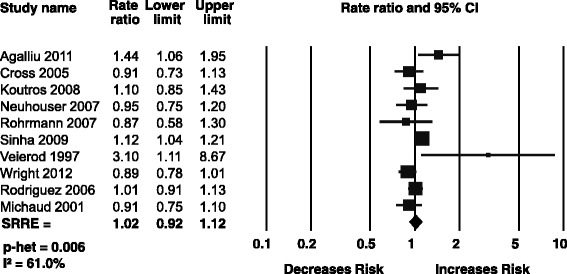
Meta analysis of total red meat and total prostate cancer

Ten prospective cohort studies were included in the meta-analysis of total red meat and total prostate cancer [13, 14, 34,36, 40, 41, 44, 45, 47–48]. Red meat was typically either undefined or defined as a combination of processed and unprocessed beef, pork, and lamb. No association between total red meat intake and total prostate cancer was observed (SRRE = 1.02; 95 % CI: 0.92–1.12), although there was significant heterogeneity between studies (Fig. 2). A sensitivity analysis was performed without the study by Veierod et al. in 1997, as the estimate was a high outlier and adjusted for very few confounding factors as compared to the other studies (age at study inclusion and attained age were the only evaluated confounding factors). The SRRE did not appreciably change with the removal of this study. No dose-response relationship was evident in meta-regression analyses or categorical intake analyses, which is overall not supportive of a positive trend. Meta-analyses were also conducted for total red meat and risk of advanced, non-advanced, and fatal prostate cancer. No significant associations were observed in these analyses, nor was there significant heterogeneity present between studies. Additionally, subgroup analyses based on geographic location, number of participants, and duration of follow-up time were conducted and no effect modification by these factors was demonstrated.

### Fresh red meat

Nine cohort studies evaluated the association between fresh red meat and total prostate cancer and were included in the meta-analysis [14, 29, 32, 34, 35, 37, 39–41]. Fresh red meat was typically defined as fresh or unprocessed beef, veal, lamb, and pork. Fresh red meat was not significantly associated with prostate cancer (SRRE = 1.06; 95 % CI: 0.97–1.16), and the model was relatively homogeneous. A sensitivity analysis was conducted after the removal of Gann et al. 1994, an outlier estimate, and Mills et al. 1989, a study in which the only confounding factor adjusted for was age. The SRRE did not appreciably change after either of these studies were removed from the analysis. Sufficient data were available to stratify analyses by race; however, no significant associations were observed for White, Black, or Asian participants. In dose-response analyses, no significant risk was observed with a one-serving increase of fresh red meat, nor was there evidence of a trend in the stratified intake analyses, although a significant association was observed in the second stratum (0.25 to <0.50 servings per day). Again, this was likely an artifact due to multiple comparisons. A meta-analysis was also conducted for fresh red meat and advanced prostate cancer but no association was observed. In subgroup analyses, no remarkable patterns of associations were observed by duration of follow-up time or geographic region. However, when analyses were stratified by number of cases, the model including studies with <1000 cases had a statistically significant SRRE (1.23, 95 % CI: 1.07–1.41), while the model for studies with >1000 cases was null (SRRE = 0.99, 95 % CI: 0.93–1.05), which may have been due to selective reporting among smaller studies. Despite this, the epidemiologic evidence does not support an independent association between intake of fresh red meat and risk of prostate cancer.

### Processed meat

**Fig. 3:**
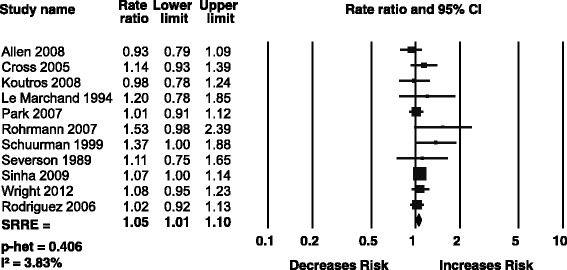
Meta analysis of processed meat and total prostate cancer

Eleven prospective cohort studies were included in the meta-analysis of processed meat and total prostate cancer [14, 30, 34, 35, 39–44, 47]. Processed meat was generally either defined as cured or salted meats, including ham, hot dogs, cold cuts, sausage, and bacon, or undefined. The result of the meta-analysis was a weakly elevated-significant summary risk estimate of 1.05 (95 % CI: 1.01–1.10) with no evidence of statistical heterogeneity between studies (Fig. 3). Analyses stratified by race showed no significant associations for White, Black, or Asian participants. In the meta-regression analysis, no evidence of a dose-response relationship was observed for incremental intake levels of processed meat. None of the stratified intake analyses produced significant associations for different categories of intake of processed meats. Sufficient data were available to perform analyses for advanced and fatal prostate cancer as well; however, no significant associations were observed in these analyses. Subgroup analyses revealed that the overall SRRE may be driven by studies conducted in the U.S. (SRRE for U.S. studies only: 1.05, 95 % CI: 1.01–1.10). Despite this, interpretation of findings for processed meat intake and prostate cancer should be tempered because of the summary association near the null value, the variability in processed meat definitions across studies, the different types of additives used in processed meat production, and the colinearity between processed meat intake and other dietary and lifestyle factors that may influence risk.

### Cooking methods and heterocyclic amines

Characteristics of prospective cohort studies of various meat cooking methods, heterocyclic amines, heme iron intake and prostate cancer are shown in Table 3 [16, 18, 34, 44, 47, 49, 50]. The results for these meta-analyses are shown in Table 4.

**Table 3:** Characteristics of prospective cohort studies of meat cooking methods, heterocyclic amines, and/or heme iron and prostate cancer ^a^

Author, year	Study cohort (Country)	No. of subjects (No. of cases)	Age range (mean)	Year baseline diet assessed	Follow-up, years	Meat cooking methods exposures analyzed	Heterocyclic amine or heme iron exposures analyzed	Prostate cancer outcome
Cross et al, 2005 [47]	PLCO (US)	29,361 (1338)	55–74	1993–2001	11	Barbequed meat; panfried meat; very well-done meat	DiMeIQx; MeIQx; PhIP; B(a)P; Mutagenic activity	Total PCa; incident PCa; advanced PCa
Jackszyn 2012 [50]	EPIC (Europe)	139,005 (4606)	>30	1989–2004	11	None	Heme iron	Total PCa, advanced PCa, localized PCa
Koutros et al, 2008 [34]	AHS (US)	23,080 (668)	48.3 [mean]	1993–1997	10	Grilled meat; pan-fried meat; broiled meat; rare or medium cooked meat; well and very well done cooked meat	DiMeIQx; MeIQx; PhIP; B(a)P; Mutagenic activity	Total PCa; incident PCa; advanced PCa
Rohrmann et al, 2015 [49]	HPFS (US)	26,030 (2770)	40–75	1996	14	None	MeIQx; DiMeIQx; PhIP; Meat-Derived Mutagenicity Index	Total PCa, advanced or lethal PCa
Sander et al, 2011 [16]	EPIC-Heidelberg (Germany)	9578 (337)	40–65	2002–2004	7	Degree of browning: strong/extreme; light/moderate	DiMeIQx; MeIQx; PhIP	Total PCa; advanced PCa
Sharma et al, 2010 [18]	Multiethnic Cohort (US)	4169 (2106)	45–75	1993–1996	8	Meat preference: medium; well done	None	Total PCa
Sinha et al, 2009 [44]	NIH-AARP (US)	175,343 (10,313)	50–71	1995–1996	8	Grilled/barbequed meat; pan-fried meat; broiled meat; microwaved meat; rare/medium cooked meat; well = done	DiMeIQx; MeIQx; PhIP; B(a)P; heme iron	Total PCa; advanced PCa; fatal PCa

^a^ PCa, prostate cancer; PLCO, Prostate, Lung, Colorectal, Ovarian Screening Trial; AHS, Agricultural Health Study; EPIC, European Prospective Investigation into Cancer and Nutrition; NIH-AARP, National Institutes of Health-American Association for Retired Persons Diet and Health Study; HFPS, Health Professionals Follow-Up Study; DiMeIQx, 2- amino-3,4,8-trimethylimidazo[4,5-f]quinoxaline; MeIQx, 2-amino-3,8-dimethylimidazo[4,5-b]quinoxaline; PhIP, 2-amino-1-methyl-6-phenylimidazo[4,5-b]pyridine; B(a)P, benzo(a)pyrene

**Table 4:** Summary relative risk estimates (SRRE), 95 % CI, *p*-values for heterogeneity and I^2^ values for cooking methods and HCA and prostate cancer (high vs. low exposure)

Model (number of studies)	SRRE	95 % CI	*P*-value for Heterogeneity; I^2^
Cooking methods			
Total Prostate Cancer:			
Grilled/Barbequed meats (*n* = 3)	1.06	0.94–1.20	0.171; 45.5 %
Pan-fried meats (*n* = 3)	1.00	0.96–1.05	0.763; 0.00 %
Broiled meats (*n* = 2)	1.04	1.00–1.09	0.451; 0.00 %
Well-done/Very well done meats (*n* = 5)	1.09	0.95–1.26	0.062; 55.4 %
Rare/Medium meats (*n* = 4)	1.01	0.91–1.12	0.245; 27.9 %
Advanced Prostate Cancer:			
Grilled/Barbequed meats (*n* = 3)	1.19	0.94–1.51	0.181; 41.5 %
Pan-fried meats (*n* = 3)	0.97	0.77–1.22	0.140; 49.2 %
Broiled meats (*n* = 2)	1.00	0.87–1.14	0.830; 0.00 %
Well-done/Very well done meats (*n* = 4)	1.24	0.90–1.71	0.075; 56.5 %
Rare/Medium meats (*n* = 3)	1.09	0.88–1.34	0.331; 9.57 %
Heterocyclic amines			
Total Prostate Cancer:			
DiMeIQx (*n* = 5)	1.03	0.97–1.09	0.530; 0.00 %
MeIQx (*n* = 5)	1.06	0.96–1.16	0.198; 33.5 %
PhIP (*n* = 5)	1.05	0.96–1.14	0.211; 31.6 %
Sensitivity analysis including “PhIP from red meat from Rohrmann 2015”	1.07	0.96–1.20	0.073; 53.4 %
B(a)P (*n* = 3)	1.01	0.90–1.14	0.169; 43.8 %
Mutagenic activity (*n* = 3)	1.09	1.00–1.20	0.725; 0.00 %
Advanced Prostate Cancer:			
DiMeIQx (*n* = 5)	1.10	0.94–1.29	0.747; 0.00 %
MeIQx (*n* = 5)	0.93	0.78–1.11	0.931; 0.00 %
PhIP (*n* = 5)	0.97	0.83–1.14	0.668; 0.00 %
Sensitivity analysis including “PhIP from red meat from Rohrmann 2015”	1.05	0.86–1.30	0.192; 34.3 %
B(a)P (*n* = 3)	1.00	0.74–1.36	0.074; 61.9 %
Mutagenic activity (*n* = 3)	1.04	0.84–1.28	0.412; 0.00 %

Five prospective cohort studies provided estimates for various meat cooking methods and consumption preferences associated with total and advanced prostate cancer [16, 18, 34, 44, 47]. Though the methods of cooking meat varied between studies, the exposure variables were categorized into five groups: broiled meats, grilled or barbequed meats, pan-fried meats, rare/medium cooked meats, and well-done/very well-done meats. All of the summary associations included the null value of 1.00 in the confidence interval. In the meta-analyses for advanced prostate cancer, no significant associations were observed.

**Fig. 4:**
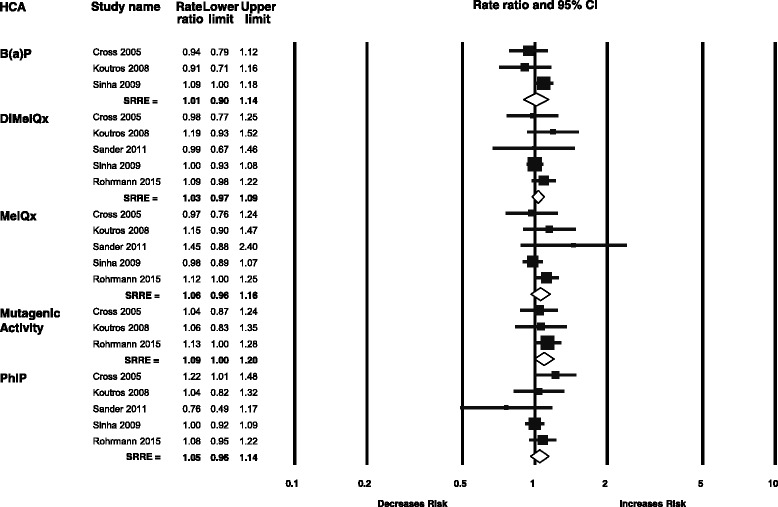
Meta-analysis of heterocyclic amines and total prostate cancer

The prospective cohort studies that provided results for cooking methods and total and advanced prostate cancer also provided estimates for various heterocyclic amines [16, 18, 34, 44, 47]. Additionally, Rohrmann et al. recently published an update of HCA consumption and prostate cancer in the Health Professionals Follow-Up Study (HPFS) cohort [49]. The heterocyclic amines evaluated included B(a)P, DiMeIQx, MeIQx, PhIP, and total mutagenic activity. No statistically significantly increased risks for total prostate cancer were observed for any heterocyclic amine (Fig. 4). The SRRE for total mutagenic activity was 1.09 (95 % CI: 1.00–1.20), mainly driven by results from the HPFS study. Similarly, in the meta-analyses for advanced prostate cancer, no significant associations were observed. The HPFS study included two estimates for PhIP intake: total PhIP and PhIP from red meat only. Because the other cohort studies used a total PhIP estimate in their analyses, we utilized that estimate from Rohrmann et al. 2015 and performed a sensitivity analysis including the PhIP estimate from red meat alone. The sensitivity analysis did not meaningfully change the SRRE from the original results. We elected not to perform dose-response analyses for cooking methods or HCA intake as no significant SRREs were observed for the high vs. low analyses, and because of the fact that HCAs are secondary exposures.

### Heme iron

Two studies were identified that included data on heme iron consumption and prostate cancer [44, 50]. No association was found for total prostate cancer (SRRE = 1.00, 95 % CI: 0.84–1.19; p-het = 0.006), and a non-statistically significant association was observed for advanced prostate cancer (SRRE = 1.06, 95 % CI: 0.73–1.55; p-het = 0.018) with statistically significant heterogeneity in both models.

### Publication bias

**Fig. 5:**
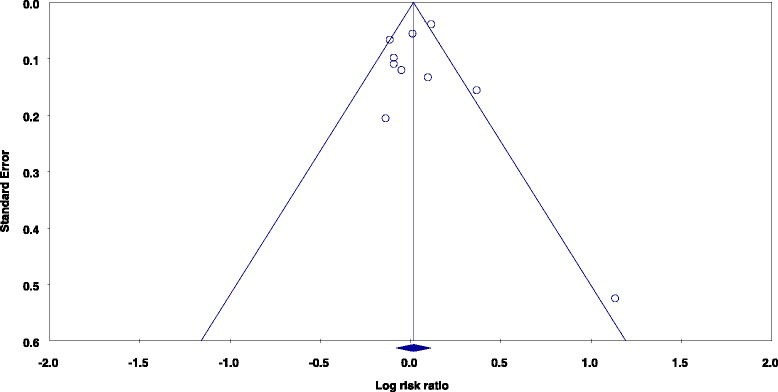
Funnel plot of prospective studies of total red meat intake and total prostate cancer

**Fig. 6:**
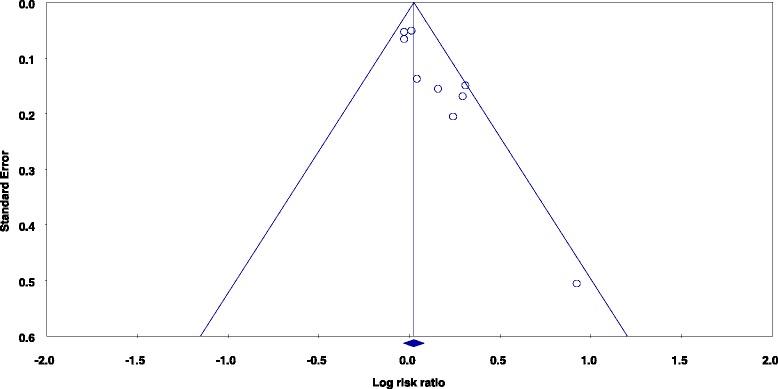
Funnel plot of prospective studies of fresh red meat intake and total prostate cancer

A visual assessment of the funnel plot for prospective studies of total red meat intake and total prostate cancer indicated the presence of slight publication bias (Fig. 5). However, Egger’s regression test was not statistically significant (*p* = 0.963) for this model. No statistical or visual evidence of publication bias was observed for studies of processed meat intake and total prostate cancer (Egger’s regression method *p* = 0.211). Of note, although marginal visual evidence of publication bias was noted for studies of fresh red meat and total prostate cancer, Egger’s regression test was statistically significant (*p* = 0.001) (Fig. 6). Duval and Tweedie’s trim and fill method was used to impute four studies to the left of the mean, resulting in an adjusted SRRE of 1.004 (95 % CI: 0.91–1.11). This SRRE was not meaningfully changed from the original SRRE of 1.06 (95 % CI: 0.97–1.16), as the confidence intervals were widely overlapped.

## Discussion

Red and processed meats have been long-debated as potential risk factors for prostate cancer. In this meta-analysis, we evaluated the association between meat and prostate cancer by quantitatively combining results from prospective cohort studies of prostate cancer and red meat, processed meat, meat cooking methods, HCA, and heme iron. In high vs. low intake analyses as well as dose-response models, we observed generally null results for the association between red and processed meat and prostate cancer.

The results from these analyses closely resemble the results from our prior meta-analysis published in 2010, although no assessment of cooking methods, HCA, or heme iron intake was undertaken in that publication [12]. No significant association was observed for red or processed meat and total or advanced prostate cancer in our previous paper, although a weakly significant association was found between a 30 g increment of processed meat and total prostate cancer (SRRE: 1.02, 95 % CI: 1.00–1.04). After updating these analyses with newly-published data from several well-established cohorts, the nature of the scientific understanding of the relationship between red and processed meat and prostate cancer does not appear to have changed substantially. Thus, the current analyses provide additional support to the notion that red meat or processed meat consumption is not an independent risk factor for prostate cancer. Of note, in our previous publication, there was some evidence of publication bias in the studies of processed meat intake and prostate cancer, a finding that was not replicated in the current analyses. The only difference between the analyses was the addition of results from the Alpha-Tocopherol, Beta-Carotene Cancer Prevention Study [14] and updated results of the NIH-AARP Diet and Health Study [44].

The presence of publication bias was observed in the studies of fresh red meat consumption and total prostate cancer. Given that the funnel plot appeared to be relatively asymmetric, it may be presumed that studies of fresh red meat and prostate cancer with negative results have not been published or reported in the scientific literature. The Duval and Tweedie imputation method was used to impute the results of four of these hypothetical studies onto the funnel plot. This theoretical exercise is used to estimate the results of a meta-analysis performed using a more even distribution of study findings. However, in this case, the resulting SRRE for the meta-analysis including the imputed hypothetical studies was not significantly or meaningfully different than the original result. Subgroup analyses for this model demonstrated that smaller studies (those with <1000 prostate cancer cases) had higher risk estimates than larger studies, an observation that is represented visually in Fig. 6. Despite the possibility of publication bias, the epidemiologic evidence that is currently available is not supportive of an association between intake of fresh red meat and prostate cancer risk.

We did not observe any increased risk specifically for advanced or lethal prostate cancer with consumption of total red meat, fresh red meat, or processed meat, or any cooking method, HCA, or heme iron. Some of the cohort studies included in our meta-analysis provided results by prostate cancer grade (high/low) [39, 49, 50]. However, these data were too sparse and heterogeneous to combine analytically. Additional prospective cohort data is necessary before any informed hypotheses can be made regarding the intake of red and processed meat and grade of prostate cancer.

Furthermore, we did not observe any overall evidence of a dose-response relationship between red or processed meat and prostate cancer in meta-regression or stratified intake analyses. However, our stratified intake analyses were based on the inclusion of RRs reported in the individual studies by each level of meat intake. For example, of the ten studies that provided an RR for total red meat consumption and total prostate cancer, only three studies included intakes of <0.5 servings per day (Veierod et al. 1997, Agalliu et al. 2011, and Koutros et al. 2008), and five data points could be included from these studies (three from Veierod et al. 1997, one from Agalliu et al. 2011, and one from Koutros et al. 2008). Consequently, the results from the stratified intake analyses may be influenced by an over-representation of certain studies at variable levels of intake, which is commonplace in nutritional epidemiology. Thus, our two different dose-response methods (i.e., linear and categorical) should serve as complimentary analyses. Future studies with more detailed quantitative intake metrics would facilitate a more comprehensive dose-response analysis with greater statistical precision and enhanced power in each intake stratum.

The 2015 HPFS update reported risk estimates for prostate cancer and HCA intake stratified by consumption of tomatoes, as lycopene is a hypothesized protective factor against prostate cancer [51, 52]. Participants consuming tomato products at least twice per week were found to have a generally decreased risk of prostate cancer when compared to those who consumed tomato products less than two times per week for analyses of consumption of most HCAs evaluated [49]. Although tomato consumption may be correlated with other healthy lifestyle factors, such as consumption of other fruits and vegetables and physical activity, these results present a novel and interesting method of viewing the data that may be useful in other prospective cohort studies.

Multiple case–control studies have been published on dietary risk factors, including red and processed meat consumption, and prostate cancer. A case–control study conducted in Uruguay found a significant trend with increasing consumption of red meat, beef, and lamb, but no increased risk or trend with processed meat consumption [53]. A recent study in Pakistan found an increased risk of prostate cancer with consumption of red meat (OR: 3.41; 95 % CI: 1.46–7.96) [54]. Results from the California Collaborative Prostate Cancer Study showed no association between both total red meat and processed meat and localized and advanced prostate cancer. Modestly-elevated significant associations were seen for advanced prostate cancer and pan-fried red meat, red meat cooked at high temperatures, and well-done red meat, but results for grilled red meat, oven-broiled red meat, and baked red meat were null [55]. A large case–control study in Caucasian men in Washington State observed increased risks of all prostate cancer, less aggressive cancer, and more aggressive prostate cancer with the highest tertile of red meat consumption [56]. Di Maso et al. analyzed data from a network of case–control studies in Italy and Switzerland and found no increased risk of prostate cancer with medium and high consumption of red meat or by an increase of 50 grams per day [57]. Collectively, associations across case–control studies are largely inconsistent. Furthermore, a very cautious and conservative interpretation should be made when reviewing data from case–control studies because of the well-known limitations of recall bias and selective participation in such studies.

There are numerous challenges to evaluating secondary exposures such as heme iron and heterocyclic amines in prospective cohort studies. First, virtually all epidemiologic studies included in this meta-analysis ascertained dietary information through a food frequency questionnaire (FFQ). It is not possible to ascertain respondents’ usual exposure to dietary mutagens or heme iron or heterocyclic amines from an FFQ. Thus, self-reported food data are translated into secondary exposures using various sources, such as the CHARRED database. The limitations of using such data have been well-documented. Thus, researchers are using self-reported dietary intake data (with underlying biases), and applying this information to a database that also has issues with exposure misclassification. This is a major limitation in these types of epidemiologic assessments. Second, many secondary exposures, such as HCA, are not included in food composition databases, since they are not a natural component of food and have no nutritional value. Third, measurement is complicated by reporting of consumption of mixed meat dishes or processed meats, whereby the specific level and type of meat is difficult to estimate. Fourth, exposure to the same mutagenic compounds reported in dietary studies may also result from tobacco smoke and other sources, as they may be present in the environment and ambient air [58]. Fifth, data from prospective studies is limited regarding other methods of meat preparation, including marinades, which have been suggested to decrease the potential for HCA formation on the surface of meat due to their antioxidant content [59, 60].

Despite these diverse limitations, we conducted meta-analyses for prostate cancer risk and HCA intake given the availability of data in prospective cohort studies and because to our knowledge, no meta-analysis of meat cooking methods or HCA consumption and prostate cancer has previously been published. There was some variation between studies in terms of variables used to assess cooking methods; for example, the category of “well-done/very well done meat” included the synonymous variables of “charred meat” and “degree of meat browning: strong/extreme” found in other publications. Most of the cohort studies that provided HCA data used the NCI CHARRED database to estimate the amount of HCA consumption in units of nanograms per day; however, if the underlying data sources representing food intake vary between studies, estimates of HCA exposure levels may be biased. In light of this, we combined measures of HCA exposures across studies using meta-analysis methodology. These analyses produced no statistically significant summary associations for total or advanced prostate cancer with high levels of exposure to PhIP, DiMeIQx, MeIQx, or B(a)P. Although the association between total mutagenic activity and total prostate cancer was 1.09, the estimate was based on only three cohort studies. More results from additional observational studies are needed before any definitive conclusions can be made regarding these exposures.

The most prevalent HCA found in meats cooked at high temperatures is PhIP [61, 62]. PhIP has been shown to induce prostate tumors in rodent models [63–65]. Creton et al. found that PhIP exposures comparable to dietary levels induced a rapid and transient increase in phosphorylation of the mitogen-activated protein kinase extracellular signal-related kinase pathway in the prostate cancer cell line PC-3. When the pathway was inhibited, migration of the PC-3 cells was significantly reduced. As the stimulation of this pathway is associated with the promotion and progression of neoplastic processes, these results suggested that PhIP may be a tumor initiator and promoter of prostate carcinogenesis [61]. Another mechanism was recently proposed by Glass-Holmes et al., who suggested that PhIP may disrupt the androgen receptor by binding to the ligand-binding domain and competing with dihydrotestoerone in the native binding cavity of the receptor [66]. Although some epidemiologic studies have observed significant associations or trends with PhIP consumption and prostate cancer [47, 49], our summary association calculated from the results of five well-established cohorts revealed null associations for PhIP intake and total as well as advanced prostate cancer. Null results for PhIP and prostate cancer risk have been corroborated in several case–control studies [55, 67]. Further research is necessary to determine why the results from epidemiologic studies of PhIP consumption and prostate cancer contrast those of mechanistic studies. As it stands, there is no compelling epidemiologic evidence to support a relationship between PhIP exposure and prostate cancer.

Additionally, mechanistic studies have implicated heme iron as a potential prostate carcinogen as a catalyst of oxidative reactions [11, 68]. We only identified two prospective cohorts that evaluated the association between heme iron consumption and prostate cancer. Heme iron was found to be associated with an increased risk of total prostate cancer and advanced prostate cancer in the NIH-AARP cohort, but not with fatal prostate cancer [44]. However, the EPIC cohort did not observe any risk for prostate cancer by stage or grade with heme iron consumption [50]. Thus, the findings from these cohorts were statistically heterogeneous. Because our findings do not support an association between red meat intake and prostate cancer, it is unlikely that heme iron is associated with increasing the risk of this malignancy.

## Conclusions

The results of our comprehensive meta-analysis show that red meat or processed meat consumption is not associated with increasing the risk of prostate cancer. A very weak, albeit statistically significant, summary association was observed for processed meat and total prostate cancer, although not for advanced or fatal prostate cancer. There was no evidence of a dose-response relationship between processed meat and total prostate cancer in meta-regression or stratified intake analyses; however, future studies with more detailed intake data would facilitate a more comprehensive evaluation of any possible dose-response patterns, particularly by prostate cancer sub-types, such as high-grade tumors. In addition, we did not observe any significantly elevated associations in the meta-analyses of cooking methods, heterocyclic amines, and total and advanced prostate cancer, although we did observe a weak association between total mutagenic activity and total prostate cancer, based on limited data. Heterocyclic amines and methods of cooking meat at a high temperature or until well-done have often been suggested as risk factors for prostate cancer. However, the results of these meta-analyses do not support that hypothesis. Based on the findings from this meta-analysis, and given the relatively large volume of prospective cohort studies, red meat or processed meat intake do not appear to be associated with increasing the risk of prostate cancer.

## Abbreviations

SRRE: Summary relative risk estimate
HCA: Heterocyclic amine
WHO: World Health Organization
PSA: Prostate-specific antigen
U.S.: United States
WCRF/AICR: World Cancer Research Fund/American Institute for Cancer Research
PAH: Polycyclic aromatic hydrocarbon
B(a)P: Benzo(a)pyrene
PRISMA: Preferred Reporting Items for Systematic Reviews and Meta-Analyses
RR: Relative risk
DiMeIQx: 2-amino-3,4,8-trimethylimidazo[4,5-f]quinoxaline
MeIQx: 2-amino-3,8-dimethylimidazo[4,5-b]quinoxaline
PhIP: 2-amino-1-methyl-6-phenylimidazo[4,5-b]pyridine
CI: Confidence Interval
HPFS: Health Professionals Follow-up Study
NCI: National Cancer Institute
NIH-AARP: National Institutes of Health-American Association for Retired Persons diet and health study
EPIC: European Prospective Investigation into Cancer and Nutrition
